# Supplementary material for: Colchicine pre-treatment and post-treatment does not worsen bleeding or functional outcome after collagenase-induced intracerebral hemorrhage
Source: PLoS One. 2022 Oct 18;17(10):e0276405. doi: 10.1371/journal.pone.0276405 (PMC9578626; doi:10.1371/journal.pone.0276405)
Supplement: Attachment — Submitted filename: Response to Reviews.docx [file pone.0276405.s002.docx]

**Response to Reviews:**

**We wish to thank the reviewers for their constructive comments. We have addressed their concerns as detailed below. We believe this has significantly improved our manuscript.**

Reviewer #1: The authors assessed the safety and efficacy of colchicine in a rat model of acute collagenase-induced ICH and its potential role as a neuroprotective agent following experimental ICH. Most of the limitations have already been discussed adequately. However, the manuscript might be improved through the following constructive feedback.

1. Colchicine is a known modulator of platelet function. The effect and risks of colchicine as a platelet function modulator in intracerebral (striatal) hemorrhage could be more focused and discussed.

**Response**: We appreciate your suggestion to discuss colchicine as a platelet function modulator. To date, no studies have investigated the effects of colchicine on platelet function in ICH. However, the effects of colchicine on platelet function are well known (as you mentioned) and have been considerably investigated in-vitro. Thus, we have added further discussion (page 19, lines 470-484). We have also noted that colchicine is a known modulator of platelet function to improve the accuracy of the sentence in our introduction (page 4, lines 56-57)

2. Minimizing the risk of ischemic events with anithrombotic therapy and otherwise risking a re-bleedin/progress of bleeding using antithrombotic therapy is usually hard to balance regarding the patient's treatment path. Is there any benefit of using colchicine instead of low-dose heparine or an early reonset of aspirin/clopidogrel therapy (e.g. 4 weeks after intracerebral hemorrhage) in reducing the risk of ischemic vascular events?

**Response**: For some time after an ICH, anti-thrombotics (anticoagulants and antiplatelets) are usually not administered due to re-bleeding risks. Although the net benefit of colchicine has yet to be established in this setting, the Colchicine for the Prevention of Vascular Events After an Acute Intracerebral Hemorrhage (CoVasc-ICH; NCT05159219) study is an ongoing randomized controlled clinical trial from our group testing the hypothesis that anti-inflammatory therapy with colchicine can reduce major adverse cardiovascular events following an acute ICH and attenuate ICH-related inflammatory brain injury. This information, with corresponding references were added in our revised manuscript (page 22, lines 578-590).

3. It would be an asset to have a conclusion at the end of the mauscript

**Response**: We have now revised our final paragraph to include a summary of the present findings, our conclusions, and a final explanation of the implications/relevance of this study. (pages 21- 22, lines 545-594).

Reviewer #2: This is an experimental study evaluating the effect of colchicine in a rat model of ICH. The authors evaluated safety and feasibility in their model of kollagenase induced ICH. The manuscript is well written and correctly reports findings according to the ARRIVE guidelines.
The main finding is that colchicine reduced the number of iron containing macophages in the perihemorrhagic zone; the authors then conclude that Colchicine treatment reduces inflammatory changes post ICH and may therefore constitute a therapeutic option after ICH.

Comments:

The abbreviation NDS is not introduced at first mention.

**Response**: The abbreviation NDS is now introduced at first mention (page 7, line 141): “Neurological deficit scale (NDS) and horizontal walking test scores were combined into a composite behavioural score as our primary endpoint for this experiment.”

P14/Line 302: "The average score at baseline........" - please specify which score (grimace score?)

**Response**: We have modified this sentence to specify the score. This sentence now reads “The average RGS score at baseline was 0.4 (out of a possible maximum score of 8) compared to 0.6 on day 1 and 0.7 on day 14.” (page 14, line 311)

Experiment 1: Was there a control group? If yes, please report the findings. If not, what was the rationale?

**Response**: In an effort to reduce animal numbers, we did not have a separate control group for experiment 1. Including such a control group iwas unnecessary because baseline measurements were obtained before treatment was initiated. As well, the endpoints of this experiment are all very well established (e.g., expected weight gain and normal temperature ranges). This justification has been added to the manuscript (page 6, lines 120-121: “This experiment did not include a control group as the expected parameters are well known in healthy animals, and baseline measures were obtained for reference.”).

The authors mention that colchicine may influence platelet function; is there any data about hemostasis/ platelet function in the current study? Bleeding time? Other platelet function tests?

**Response**: The effects of colchicine on blood clotting is a concern, but studies that have found significant effects on hemostasis and platelet function have mostly used doses of colchicine much higher than those that are safe to achieve clinically. Because our study used a low-dose of colchicine, specifically chosen to be safe and clinically-relevant, it is unlikely that we would have found a significant effect on hemostasis with our dosing regimen. Thus, we did not perform any tests of platelet function or assess peripheral bleeding time (e.g., tail bleed). However, any biologically-meaningful effects that colchicine on hemostasis should have been detected by group comparisons of intracerebral hematoma volume (most biologically relevant endpoint for this study), which were not significant. A similar explanation, along with additional discussion on the effects of colchicine on platelet function and aggregation, have now been added (discussion, page 19, lines 470-484).

The authors state that colchicine may have different anti-inflammatory effects, e. g. via influencing chemokine, cytokine expression, inflammatory cell proliferation and migration and has been shown to affect the NLRP3 inflammasome. In the present study they assessed PErls positive cells, i.e. cells containing iron residues as only marker for neuroinflammation. Given that drug treatment reduced this parameter I would suggest to further elaborate on the possible anti-inflammatory effect, e. g. by assessing microglia activation and other components of the inflammasome to further elaborate on this finding. This would substantially strenghten their conclusions.

**Response**: Given that colchicine did not affect any of our primary endpoints of treatment efficacy (e.g., edema) and safety (e.g., hematoma volume), we can infer that any effects on the inflammatory response were inconsequential to stroke outcome. Notably, the impact of colchicine on inflammation was not sufficient to impact brain edema, cell death, or functional recovery. Had we found positive findings on any of those key, widely-accepted endpoints, we would have investigated the inflammatory response further to document the changes and to better identify important mechanisms of action. As suggested by the reviewer, we have added text to our discussion (page 21, lines 527-537) to acknowledge this, as well as to mention those additional endpoints (e.g., cytokine and chemokine expression) that could be assessed to gain further insight into the inflammatory effects of colchicine should, for instance, other colchicine treatment regimens provide benefit against ICH in animals or patients.
